# Erratum: Ingelfinger, J.R.; *et al*. Averting the Legacy of Kidney Disease—Focus on Childhood. *Children* 2016, *3*, 4

**DOI:** 10.3390/children3020006

**Published:** 2016-04-18

**Authors:** Children Editorial Office

**Affiliations:** MDPI AG, Klybeckstrasse 64, CH-4057 Basel, Switzerland; children@mdpi.com; Tel. +41-61-683-77-34; Fax: +41-61 302-89-18

The *Children* Editorial Office wishes to notify its readers of a correction in [1]. In proofing, the abbreviation HN was changed to hypertension. HN stands for hereditary nephropathy and should have been defined as such when a legend was added in proofing. However, due to the change in the abbreviation to hypertension, hypertension was erroneously inserted in the legend.

The Table 2 should read:

The manuscript will be updated and the original will remain available on the article webpage. The authors would like to apologize for any inconvenience caused.

## Figures and Tables

**Table 2:** Etiology of chronic kidney disease in children *.

CKD Etiology	Percentage (Range)	ESRD Etiology	Percentage (Range)
CAKUT	48%–59%	CAKUT	34%–43%
GN	5%–14%	GN	15%–29%
HN	10%–19%	HN	12%–22%
HUS	2%–6%	HUS	2%–6%
Cystic	5%–9%	Cystic	6%–12%
Ischemic	2%–4%	Ischemic	2%

Rare causes include congenital NS, metabolic diseases, cystinosis/miscellaneous causes depend on how such entities are classified. CAKUT: Congenital anomalies of the kidney and urinary tract; GN: Glomerulonephritis; HN: Hereditary nephropathy; HUS: Hemolytic uremic syndrome. * from Harambat *et al.* CKD data are from NAPRTCS, the Italian Registry and the Belgian Registry. ESRD: end-stage renal disease; ESRD data are from ANZDATA, ESPN/ERA-EDTA, UK Renal Registry, and the Japanese Registry.
